# Late Recurrence of a Rare Middle Ear Neuroendocrine Tumor With an Intracranial Extension to the Temporal Fossa: A Case Report

**DOI:** 10.7759/cureus.37900

**Published:** 2023-04-20

**Authors:** Lukas Kvaščevičius, Eugenijus Lesinskas, Donatas Petroška, Robertas Kvaščevičius, Inga Šatinskienė

**Affiliations:** 1 Medicine, Vilnius University, Vilnius, LTU; 2 Otorhinolaryngology, Vilnius University Hospital Santaros Klinikos, Vilnius, LTU; 3 Pathology, Vilnius University Hospital Santaros Klinikos, Vilnius, LTU; 4 Neurosurgery, Vilnius University Hospital Santaros Klinikos, Vilnius, LTU

**Keywords:** subtemporal zone, temporal fossa, middle ear neoplasms, carcinoid, menet, neuroendocrine tumor, middle ear

## Abstract

We report a clinical case of an extremely rare neuroendocrine tumor of the right middle ear (MeNET) that recurred after 13 years with a local extension into the right temporal fossa. In the current medical literature, there are approximately 150 cases of MeNETs and even fewer cases with more than 10 years of follow-up, recurrence, and intracranial tumor progression. Therefore, we believe that this paper can make an important contribution to the existing and future knowledge about this disease. The purpose of this article is to present our experience in treating such a rare neoplasm in a 35-year-old woman. The patient initially complained of worsening hearing in her right ear over the past year. The final diagnosis was made based on the findings of computed tomography (CT), magnetic resonance imaging (MRI), and histological and immunohistochemical evaluation of excisional biopsies of the original and recurrent tumors. The primary tumor masses were removed with clear resection margins, and the ossicular chain was reconstructed. The patient has been monitored clinically and radiologically with temporal bone CTs every year and MRIs three times in general since then. A postoperative audiogram showed remaining mixed hearing loss in the right ear that eventually worsened as the tumor grew. Tumor recurrence and progression after 156 months (13 years) were seen on CT and MRI, requiring further treatment. After resection of the recurrent tumor, paresis of the right facial nerve developed, which was treated with dexamethasone. The surgical treatment caused the initial symptoms to disappear, but the facial nerve paresis persisted with mild functional improvement. The patient is not receiving adjuvant radiotherapy and is being monitored closely because the tumor may recur in the future.

## Introduction

Middle ear neuroendocrine tumors (MeNETs) are an extremely rare clinical entity representing <2% of primary ear tumors [1]. To our knowledge, there are around 150 published cases of MeNETs in the current medical literature and even fewer reports of recurrence and invasion into surrounding structures [2]. The first to describe a distinct glandular middle ear neoplasm from paraganglioma and salivary gland tumors were Hyams and Michaels in 1976 [3]. Since then, many names have been used over the years, such as middle ear adenoma, adenomatous neuroendocrine tumor, and carcinoid or neuroendocrine carcinoma, but, in general, neuroendocrine tumors (NETs) could be regarded as an umbrella term for this entity. According to the updated 2022 World Health Organization (WHO) classification of head and neck tumors, previously mentioned synonymous terminology is not recommended, and middle ear neuroendocrine tumor (MeNET) is the preferred term now, but having coding assignations as malignant by the International Classification of Diseases for Oncology (ICD-O): 8249/3 (neuroendocrine tumor) and ICD-11: 2C21.Y & XH8DS0 (other specified malignant neoplasm of middle ear & neuroendocrine tumor, NOS) [4]. The unifying theory is that these tumors are associated with undifferentiated pluripotent stem cells derived from the neural crest and share a common phenotype and immunohistochemical profile with neuroendocrine differentiation [5]. Classification, etiology, and biological characteristics are poorly understood. According to published clinical cases, about 50-year-old patients (ranges between 14 -80 years old) without any gender predilection are usually affected [5,6]. In this article, we present a case of MeNET, which relapsed 156 months (13 years) after an original surgical removal with clear resection margins and extended into the temporal fossa. We also review diagnostical imaging, histological, and immunohistochemical findings and discuss treatment as well as follow-up strategies.

## Case presentation

**Figure 1:**
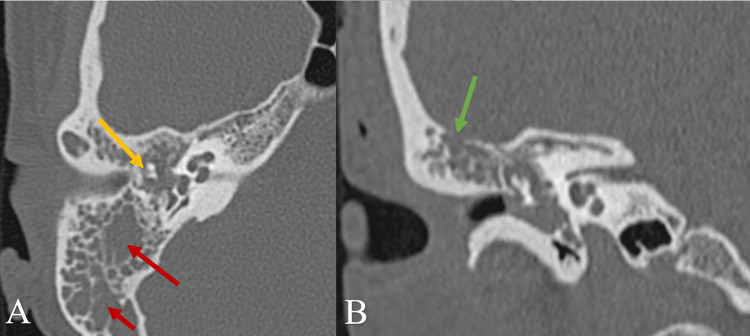
Preoperative axial (A) and coronal (B) CT images of the right ear in 2009 Opacification of the mastoid air cells (red arrows), ossicles embedded but not destroyed by the tumor (yellow arrow), and dehiscence of tegmen mastoideum (green arrow) are seen.

In 2009, a 35-year-old woman presented to the Department of Otorhinolaryngology of Vilnius University Hospital Santaros Klinikos with reduced hearing in her right ear that had worsened over the past year. From the patient's medical history, recurrent otitis media was documented. The otoscopy revealed a hard, rough, inert, painless, and non-bleeding mass in the frontal part of the eardrum protruding through the tympanic membrane from the tympanic cavity (epitympanum). Tympanogram of the right ear (AD) and left ear (AS) showed B/A type curves, respectively. Rinne and Weber tests indicated conductive hearing loss on the affected side: Rinne test AD/AS was negative/positive, respectively; Weber test showed lateralization to the right. The pure tone audiogram in the right ear showed mixed hearing loss with mean hearing loss 64±5,2 decibels (dB), air-bone gap 39,5±4,2 dB, and bone conduction threshold 24,7±8,5 dB of hearing level. The audiogram in the left ear was normal. Middle ear computed tomography (CT) showed masses in the epitympanum (HU - 23-100), opacification of the mastoid air cells, ossicles embedded by the tumor, and dehiscence of the tegmen mastoideum (Figure 1). At that time, the differential diagnosis included pus and chronic otitis media. The operation was chosen as a tool for excisional biopsy and a more detailed intraoperative evaluation of the damaged surrounding structures by the tumor. A canal wall-up mastoidectomy of the right ear using the retroauricular approach was performed, and the tumor was subtotally removed. Tumor masses were histologically and immunohistochemically verified as low-grade (grade 1) neuroendocrine tumors.

**Figure 2:**
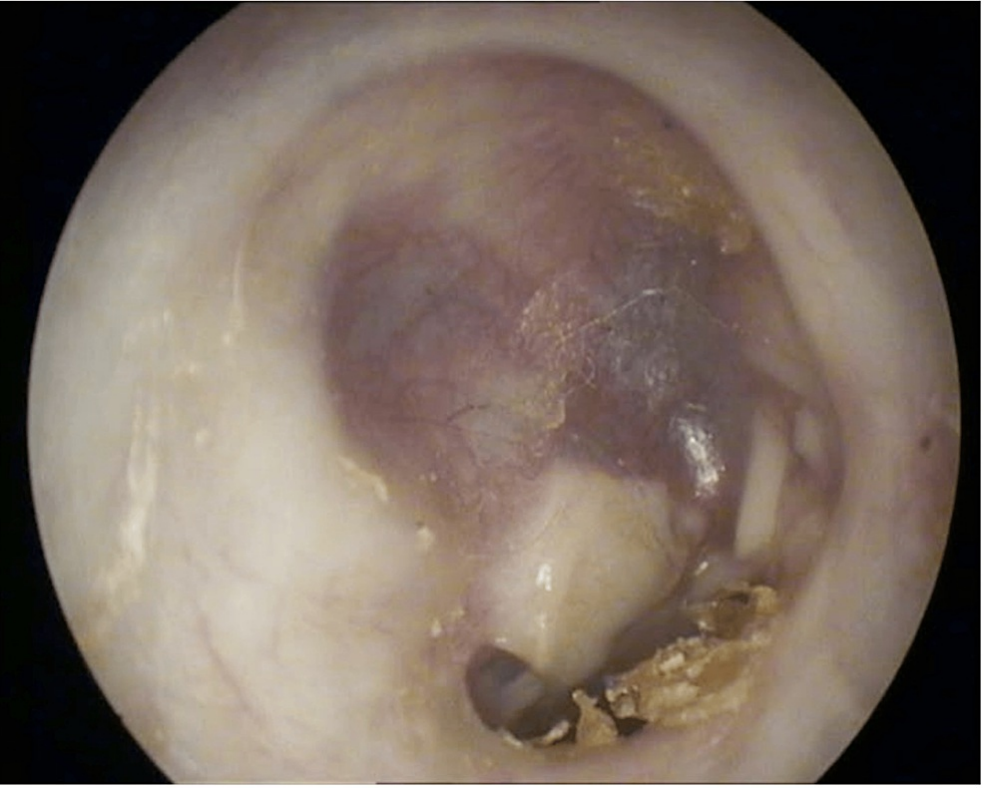
Otoscopic view of the right ear, 12 months after the second surgery. No signs of tumor local recurrence are seen. The lateral attic wall is covered with the fascia to detect early relapse of the tumor

**Figure 3:**
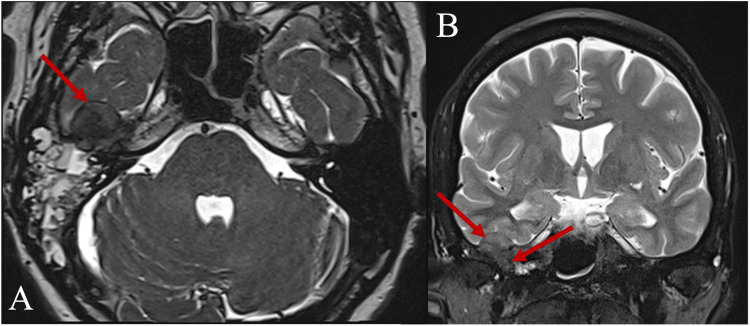
Head MRI before the latest surgery, T2-weighted axial (A) and coronal (B) views Tumor recurrence is seen. Arrows show intracranial infiltration into the dura mater of the temporal fossa (~18 mm in length and ~8 mm in width segment) and subtemporal extension of the tumor. Clear invasion into brain parenchyma or any compression of its structures is not observed.

**Figure 4:**
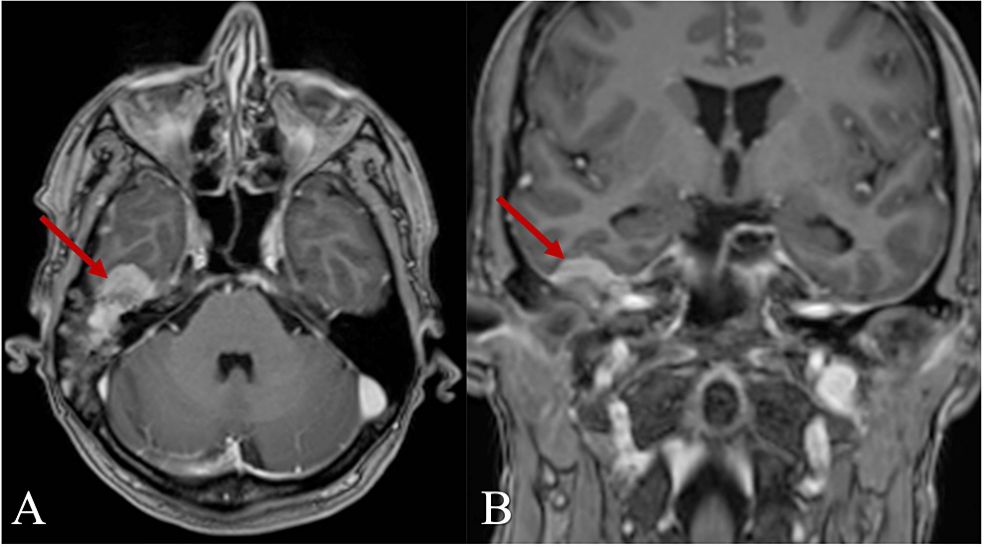
Head MRI of the relapsed tumor, T1-weighted axial (A) and coronal (B) views Arrows indicate a contrast-enhancing tumor extending intracranially into the temporal fossa and subtemporally.

**Figure 5:**
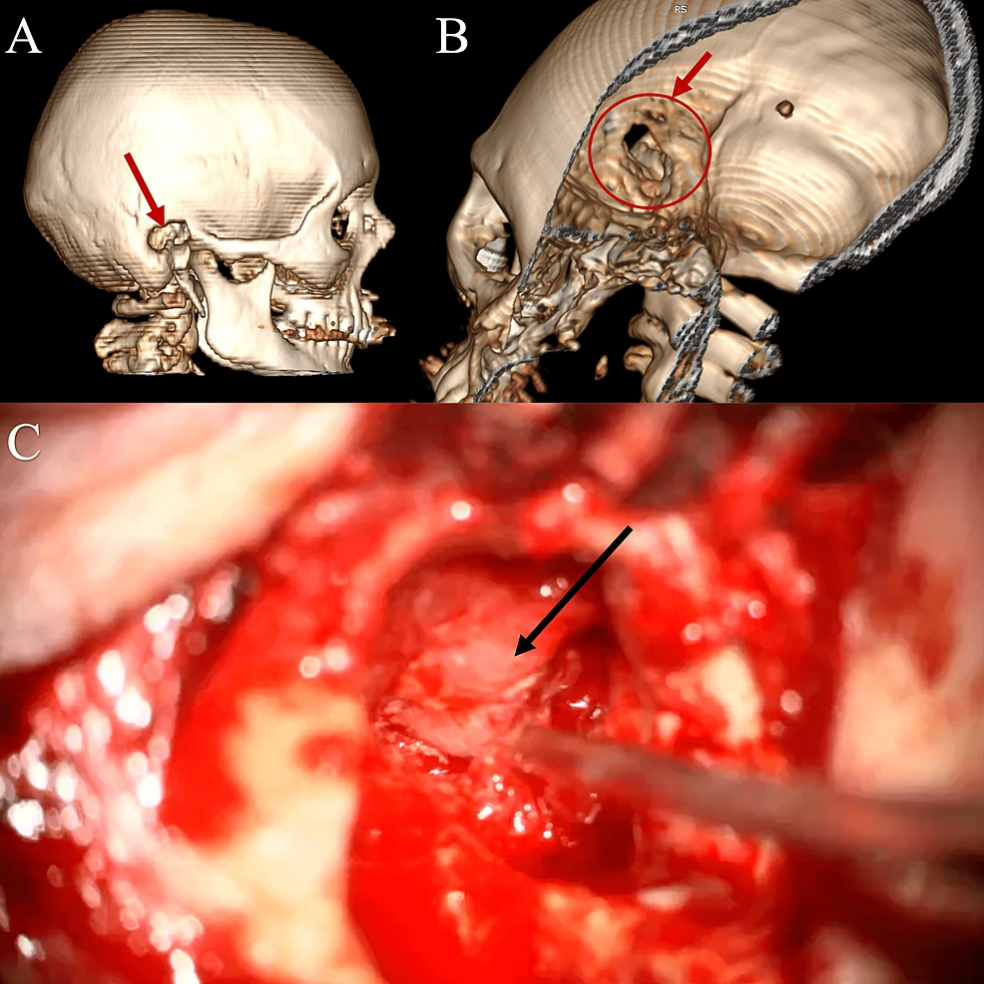
3D cranial bone reconstruction of re-mastoidectomy (red arrows and circle), lateral (A) and medial (B) views. Intraoperative field of vision (C) during the most recent surgery The tumor (black arrow) looks pinkish, hypervascular (bleeding), and strongly attached to the dura mater.

In 2011, a residual tumor in the same location was treated with canal wall down mastoidectomy and complete resection together with the ossicular chain reconstruction. In more detail, during surgery, tumor masses were found after opening the mastoid cells. A tumor with an extension to the anterior epitympanum and especially to the pyramidal process of the petrous temporal bone, involving the antrum, the tympanic cavity, the tympanic sinus, and the hypotympanic peritubal space was resected. Eroded malleus head and incus were removed. The stapes were intact. There was no evidence of bony erosion of the facial canal or the labyrinth. Ossiculoplasty with a titanium partial ossicular replacement prosthesis (PORP-type) and tympanoplasty using both the conchal cartilage and the temporal fascia were performed. The lateral attic wall was covered with the fascia to detect early relapse of the tumor (Figure 2). There were no perioperative complications, and the patient was followed clinically and radiologically with temporal bone CTs every year and MRIs three times in general since then. After the second operation, mixed hearing loss remained in the right ear. On the audiogram, average hearing loss decreased to 90 decibels over the period of 11 years. Close monitoring was discontinued during the coronavirus disease 2019 (COVID-19) pandemic until safety restrictions allowed us to resume diagnostics. In 2022, 13 years after the initial treatment, the same patient presented to the outpatient clinic complaining of non-specific wet discharge from her right ear and aural fullness in the right ear that had worsened over the last four months. Head magnetic resonance imaging (MRI) was performed and revealed heterogenic tumor masses (~33 x 20 x 23 mm in size) with protein-like fluid content and contrast-enhancing zones. Intracranial infiltration into the middle cerebral fossa was seen (Figures 3, 4). The last two temporal bone CTs and MRIs seven years apart confirmed tumor recurrence and progression. The patient was admitted to the Department of Neurosurgery, where re-mastoidectomy, as shown in Figure 5, and tumor resection with duroplasty were performed. Intraoperatively, tumor masses looked pinkish, slightly bleeding (hypervascular), and strongly attached to the dura mater of temporal fossa (Figure 5). The affected bone around the tumor was resected, and piecemeal tumor removal with negative resection margins was performed. The infiltrated site of the dura was coagulated, excised, and reconstructed using transplanted fascia and adipose tissue from the abdominal wall with fibrin glue fixation.

**Figure 6:**
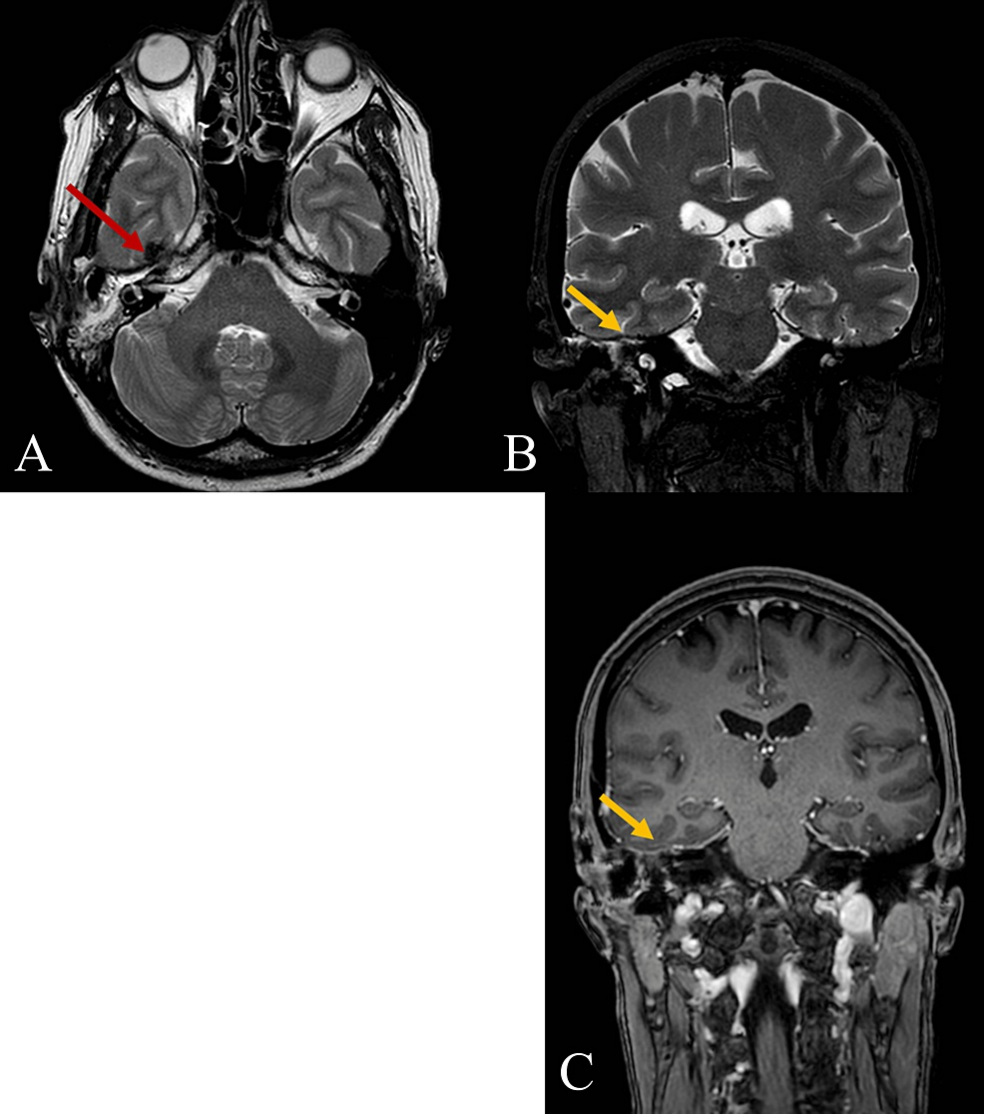
Postoperative MRI in T2-weighted axial (A), coronal (B) and T1-weighted coronal (C) views, the latest follow-up in 2023 Postoperative changes (red arrow) after the surgical tumor removal and duroplasty is seen. No residual tumor in the previously affected location (yellow arrows) is visible.

A right facial nerve paresis developed after the surgery (according to the House-Brackmann grading system - 4 points) which was treated with dexamethasone with only mild improvement of the facial nerve function. There were no other postoperative complications, such as leakage of cerebrospinal fluid (CSF). During a tumor board meeting, a general consensus was reached that adjuvant radiotherapy was not recommended for the particular case. The latest MRI follow-up was performed in 2023, which showed no residual tumor in the right middle ear and around the dura of the temporal fossa (Figure 6). The most recent consultation with the patient revealed that the patient's initial symptoms regressed, and facial nerve palsy slightly improved over the period of four months.

Histological and immunohistochemical results

**Figure 7:**
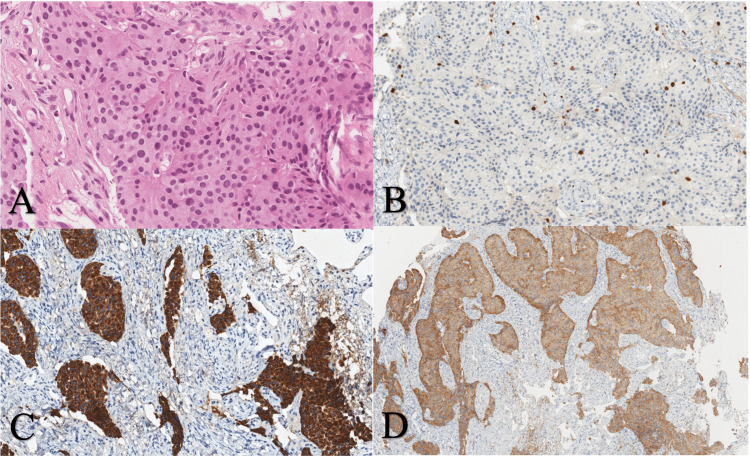
Hematoxylin and eosin staining of initial tumor tissue at 800x magnification shows merged solid components with eosinophilic cytoplasm, round nuclei, and no mitosis. (A) Positive Ki67 expression in few cells (B), and strongly positive PanCK expression in all tumor cells (C) are shown at 400x magnification. Moderate to strong expression of synaptophysin (D) at 200x magnification Ki67 - marker of proliferation Ki67, PanCK - pancytokeratin

**Figure 8:**
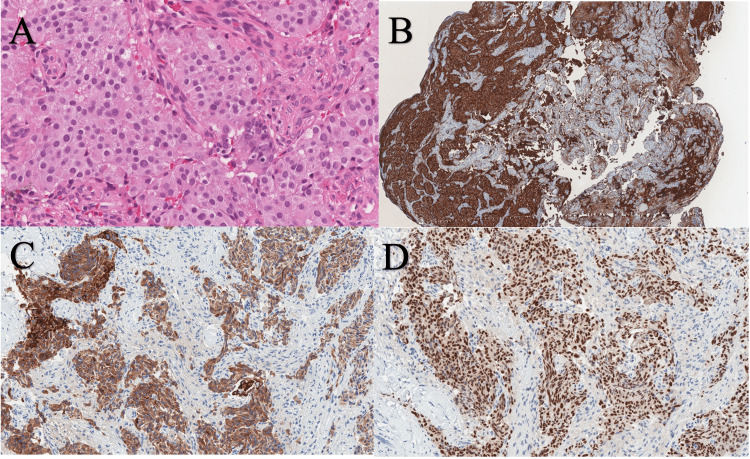
Hematoxylin and eosin staining of the recurrent tumor tissue at 800x magnification shows small cells with eosinophilic cytoplasm, round nuclei, and no mitosis merged into groups, dynamically without change (A). Strongly positive expression of chromogranin A at 100x magnification (B), Cam5.2 (C) and Islet1 (D) markers at 400x magnification is depicted Islet1 - insulin gene enhancer protein ISL-1

Upon the removal of the tumor, segments of the mass were sent for histopathological and immunohistochemical evaluation. Histology of primary and recurrent tumors revealed fibrous vascularized tissue merged with solid components composed of small cells with eosinophilic cytoplasm and round, monotonous nuclei. No mitosis or necrosis was seen. Immunohistochemical results showed highly positive cytoplasmic reaction of epithelial markers Cam5.2, PanCK and neuroendocrine markers Islet 1 (ISL1), synaptophysin (Syn), chromogranin A (CgA) in 100% of tumor cells; Ki-67 proliferation index was estimated as 1-5% of tumor cells (Figures 7, 8). Serotonin was expressed in some cells, S-100 and TTF1 markers were negative (these markers are not presented in figures).

The first biopsy 13 years ago verified a well-differentiated grade 1 neuroendocrine tumor (carcinoid), and it showed a low Ki67 index (2-3%), absence of necrosis, and no mitotic activity. In 2022, the excisional biopsy of the relapsed tumor for re-evaluation was performed, which clarified it to be a grade 1 MeNET according to the latest nomenclature of the 2022 WHO classification of head and neck neuroendocrine neoplasms [4]. 

## Discussion

Diagnosis of MeNETs is often challenging since clinical and radiological findings are unspecific. The most common and usually the main symptom is progressive unilateral (conductive or mixed) hearing loss [2]. Other clinical manifestations such as blockage, aural fullness, otalgia, tinnitus, or vertigo may occur, depending greatly on the size, location, and infiltration of the tumor. Facial nerve paresis has also been reported, which could be more related to anatomical abnormalities or local compression rather than an invasion of the tumor [7-9]. Differential diagnosis of middle ear masses with progressive hearing loss includes middle ear otitis, cholesteatoma, acoustic neuroma (schwannoma), meningioma with glandular structure, encephalocele, rhabdomyosarcoma, jugulotympanic paraganglioma, endolymphatic papillary tumor, and adenocarcinoma. As the list of possible causes is extensive, radiographic, histologic, and immunohistochemical findings in combination help to distinguish MeNET from other entities [5,6].

In clinical practice, one should think about these tumors when the patient presents with chronic otitis media, facial nerve palsy, and conservative treatment is ineffective [10]. Radiological work-up consists of temporal bone CT for bone invasion or destruction and MRI for more detailed visualization of soft tissues. In our case, the patient had recurrent otitis media, the first preoperative temporal bone CT suggested chronic otitis media, and further surgical intervention was required to clear this suspicion. In some cases, single-photon emission computerized tomography (SPECT) or positron emission tomography (PET) scans can be used as a diagnostic tool for metastasized disease [9]. The imaging features are non-specific and still relatively ill-defined since they are based on scarce case reports. Typical CT images include a soft tissue mass in the mastoid bone without additional blood vessels, which may extend to the middle ear and mastoid. Bone remodeling and expansion are also common findings [5]. The ossicular bones are usually embedded within the tumor without any bone erosion [6]. On MRI, MeNET is isointense to hyperintense and contrast-enhancing in T1-weighted images, whereas in T2-weighted images, it is approaching the intensity of the gray matter signal [6]. Macroscopically, the tumor is white, grey, or red-brown, with a soft or rubbery consistency. On histopathological view, MeNETs typically exhibit cuboidal to columnar cells with distinct cell borders and eosinophilic cytoplasm. Nucleoli can rarely be found in the small central hyperchromic nuclei. The tumor's appearance shows many patterns with stromal infiltration, including a glandular configuration in a fibrous stroma, back-to-back, pseudorosette, trabecular, and sheet-like patterns, along with discohesive single cell infiltration; many have prominent swirling of tumor cell ribbons. Mucins can be demonstrated within the gland lumen. Some tumors show significant nuclear pleomorphism and increased mitotic activity [4]. Our histological findings included aggregated small cells with eosinophilic cytoplasm and round nuclei.

MeNETs usually express many various epithelial and neuroendocrine markers, such as cytokeratin 7 (CK7), CgA, neuron-specific enolase (NSE), Syn, human pancreatic polypeptide (HPP), CAM5.2, EMA, CD56, S-100, LEU-7, serotonin, glucagon, adrenocorticotropic hormone, somatostatin, various polypeptides as well as transcription factors. CK7 and ISL1 markers are found to be distinctively positive in neuroendocrine tumors [5,7,11,12]. Our immunohistochemical results showed strongly positive PanCK7, ISL1, CAM5.2, CgA, synaptophysin markers, and serotonin expression and were negative for both S-100 and thyroid transcription factor (TTF) markers. These findings were consistent with neuroendocrine tumors in the existing literature and differentiated MeNET from other tumors, such as paraganglioma, the most common tumor in the middle ear, but immunohistochemically should be negative for keratins and have sustentacular cells positive for S100. The most recent reviews do not suggest any reliable, based on consensus, histopathological or immunohistochemical markers that predict tumor behavior and overall clinical outcome; therefore, a long-term follow-up is recommended [9].

Current MeNET classification is incomplete; however, historically, two large studies have suggested ways to classify MeNETs: one according to immunohistochemical markers and the presence of metastasis and another based on TNMS. In 2009, Saliba et al. in their study classified middle ear glandular tumors (neuroendocrine adenomas) into three types [8]. Another more recent TNMS classification was presented by Marinelli et al. in a large multi-national study in 2018. It resembles the classic TNM classification where T stands for tumor, N for nodular component, M for distant metastasis, and adds S - a component that means presence or absence of secretion [2]. Following this latest tumor grading system, our case could be regarded as T3 since the tumor invaded the dura without any nodular or other distant metastasis and pathological secretion (T3, N0, M0, S0 disease). 

To date, surgery and radical removal of the tumor seem to be the treatment of choice. However, there is no consensus on the optimal surgical method: some medical experts favor the most radical approach possible, while others support a more conservative way. Ultimately, our goal was to evaluate the benefit-risk ratio of the procedure for the patient and maintain the balance between functional results (relieving neurologic symptoms, improving hearing) and the most possible radical removal of the tumor. The approach of intervention mostly depends on the size, location, and extension of the tumor. A transcanal approach is suitable for small tumors which are limited to the mesotympanum or masses that protrude through the tympanic membrane into the outer ear canal. Larger tumors that extend to the epitympanum and/or the mastoid can be removed through a tympanoplasty and intact canal wall mastoidectomy. The recurrence rate is slightly higher with a transcanal tympanoplasty (14%) compared to radical mastoidectomy (9%); however, every case is individual, and the superiority of one procedure over the other cannot be determined [6,13].

Petrosectomy, a more extensive procedure, could be a viable option in the radical treatment of recurrent middle ear tumors that extend beyond the tympanomastoid space. It is typically reserved for patients with recurrent chronic otitis media, cholesteatoma, and malignant tumors such as squamous cell carcinoma (SCC) of the temporal bone. This procedure is performed on ears with complete hearing loss that requires multiple surgeries [14]. However, there is insufficient knowledge about the usage and effectiveness of petrosectomy in patients with middle ear tumors (particularly MeNETs). It is believed that MeNETs are low-grade malignant lesions with benign cell morphology that locally reoccur in 15-25 % of cases and metastasize in 7-9% of cases (mostly to cervical lymph nodes) [15,16]. The mean follow-up time published before 2018 was approximately 55 months. The most recent and larger study of nine patients by van der Lans et al., with a median of 155 months follow-up time, proposed that an even higher percentage (71%) of patients had recurrent MeNET after being treated for local and non-aggressive tumor [9]. However, in this article, recurrence and invasion into the dura mater of the middle cranial fossa were not mentioned. These findings suggest that the previously assumed relatively benign course of MeNET does not always happen. It could be regarded as a tumor with a potentially malignant clinical behavior since it is capable of progressing beyond tympanomastoid area many years later. Following this logic, a more radical approach could favor the patient in order to reduce the risk of recurrence and prevent multiple surgeries in the future. Lateral petrosectomy (according to Dr. Ugo Fisch "subtotal") involves a resection of the complete outer canal, the eardrum and a portion of the mastoid lateral to the labyrinth. Subtotal (according to Dr. U. Fisch "total") petrosectomy extends from the carotid canal to the jugular fossa and includes transection of the petrous bone medial to the labyrinth and through the internal auditory canal. Total petrosectomy extends further and involves the petrous apex. The cochlear nerve is sacrificed, and the facial nerve can be easily harmed in subtotal and total petrosectomies [17]. For that reason, the subtotal and total petrosectomy approach seemed too radical for us since we wanted to preserve and improve hearing for the patient as well. According to a large retrospective study presented by Prasad et al., subtotal (lateral) petrosectomy has been suggested to provide better results than canal wall down mastoidectomy alone in terms of postoperative recurrence (1.1 % and 1.5-17%, respectively) and minimal risk of postoperative complications in various situations [14]. Therefore, lateral petrosectomy could be considered in our case since it offers radical removal of the tumor, a relatively low incidence of complications, and preserves important surrounding structures (vestibulocochlear and facial nerves, dura mater), which in combination keeps the balance between functional and tumor control effects. 

When the ossicular chain is involved, complete resection and later reconstruction of it bring more favorable results compared to subtotal resection of the tumor with preservation of intact ossicular bones due to higher recurrence rates in latter cases [7,8]. However, uncertainty arises when contemplating the overall significance of ossiculoplasty in relation to middle ear tumors. Due to the rarity of middle ear tumors, most of the data is orientated to chronic middle ear diseases (chronic otitis media, cholesteatoma) and their relation between functional and disease control outcomes after the ossicular reconstruction. Therefore, the question of reliable and long-term data on a clear connection between the non-reconstruction of the ossicles and a decrease in the frequency of middle ear tumor recurrence is open for discussion. In our case, for the initial MeNET, a combination of both canal wall up and canal wall down mastoidectomies with ossicular chain reconstruction was performed with the intention to radically remove visible tumor masses and establish good local control. On the other hand, the tumor relapsed, and the ossiculoplasty gave little improvement in our patient's hearing rehabilitation. In a recent large study, reconstruction of the ossicles using titanium prosthesis resulted in postoperative mean air-bone gap ≤20 dB in 65% of cases, with better results in partial compared to total ossiculoplasty. It is worth mentioning that the study was mostly orientated to patients with chronic otitis media and cholesteatoma, and only three out of 256 patients were treated for tumor masses (not specified) [18]. Practically, if we fail to restore or improve hearing in the affected ear by means of the ossicular chain reconstruction, then performing an extensive radical operation (such as lateral or subtotal petrosectomy) and avoiding ossiculoplasty could potentially give better control of the tumor in the long run. By totally removing all the middle ear structures (particularly the ossicles) we are ensuring that no residual tumor cells remain and the risk of recurrence in the future is reduced to a minimum. As for auditory rehabilitation, alternative options are possible, such as bone anchored hearing aid (BAHA), vibrant soundbridge (VSB), or cochlear implantation (CI) with adequate functional gain [19].

For the relapsed tumor, re-mastoidectomy, total tumor resection from bone structures, and the dura mater of the temporal fossa with duroplasty were achieved. Surgical methods and adjuvant radiotherapy were discussed in a tumor board meeting. Neurosurgical intervention was needed since the recurrent tumor involved the dura mater and carried a higher risk of intraoperative and postoperative complications. As discussed with expert oncologists, our patient did not receive any radiotherapy or chemotherapy. To date, the limited literature and clinical practice show poor results in terms of inadequate tumor response to adjuvant treatment and overall patient survival (damage and fibrosis of surrounding healthy tissue, which may lead to secondary malignancy). Radiotherapy or chemotherapy should be used only in complicated cases with distant metastases. Further studies on its efficacy are needed [7,16,20].

Our patient did not present with any facial nerve dysfunction; however, facial nerve paresis appeared postoperatively as a complication, and we think that it happened for a couple of reasons. The first reason being direct damage or compression of the tumor, as it has been reported in a recent large study where 16% of patients (five out of 32 cases) had facial nerve dysfunction due to direct dense adhesion and/or invasion of the tumor [2]. The second reason being an already uncovered facial nerve due to extensive manipulations in the patient's right ear over the course of long treatment. Among mentioned reasons, postoperative facial nerve edema might also cause nerve dysfunction. However, dexamethasone, as a standard method of treatment, gave relatively little and slow improvement of the facial nerve paresis. Prevention of it is a matter of debate, but the patient was informed about the possible risks before each operation. In general, the reported overall five and 10-year survival rate in MeNET patients is positive, with 94% and 92%, respectively [16]. While our article highlights a rare occurrence of dura mater infiltration in the temporal fossa by the MeNET after complete tumor resection and a median follow-up time of 156 months (13 years), it is important to recognize that this is constrained by the limited scope of a single case. 

## Conclusions

We have reported a case of rare MeNET, which recurred after 156 months (13 years) since a surgical removal with clear resection margins and extended into the dura mater of the temporal fossa, suggesting its uncertain (or rather malignant in terms of progression) clinical behavior. In almost any case, the treatment of choice for local tumors has been suggested to be a complete surgical excision, preferably with negative resection margins. The prospect of the disease course is hard to define and may vary individually. Since long-term recurrence is possible after the initial successful surgical treatment, these patients should be inspected regularly for any signs of progression.
